# Comparison of the Single Cell Immune Landscape between Subjects with High *Mycobacterium tuberculosis* Bacillary Loads during Active Pulmonary Tuberculosis and Household Members with Latent Tuberculosis Infection

**DOI:** 10.3390/cells13040362

**Published:** 2024-02-19

**Authors:** Supitcha Kamolratanakul, Wassawon Ariyanon, Kanyarat Udompornpitak, Thansita Bhunyakarnjanarat, Asada Leelahavanichkul, Jittima Dhitavat, Polrat Wilairatana, Wiwat Chancharoenthana

**Affiliations:** 1 Department of Clinical Tropical Medicine, Faculty of Tropical Medicine, Mahidol University, Bangkok 10400, Thailand; supitcha.kam@mahidol.edu (S.K.); jittima.dhi@mahidol.ac.th (J.D.); polrat.wil@mahidol.ac.th (P.W.); 2 Tropical Immunology and Translational Research Unit (TITRU), Department of Clinical Tropical Medicine, Faculty of Tropical Medicine, Mahidol University, Bangkok 10400, Thailand; 3 Department of Medicine, Banphaeo General Hospital (BGH), Samutsakhon 74120, Thailand; jowassawon@gmail.com; 4 Department of Microbiology, Faculty of Medicine, Chulalongkorn University, Bangkok 10330, Thailand; jubjiibb@hotmail.com (K.U.); thansitadew@gmail.com (T.B.); aleelahavanit@gmail.com (A.L.); 5 Center of Excellence on Translational Research in Inflammation and Immunology (CETRII), Department of Microbiology, Faculty of Medicine, Chulalongkorn University, Bangkok 10330, Thailand

**Keywords:** CyTOF, latent tuberculosis, pulmonary, responder, tuberculosis

## Abstract

It is unclear how the immune system controls the transition from latent tuberculosis (TB) infection (LTBI) to active pulmonary infection (PTB). Here, we applied mass spectrometry cytometry time-of-flight (CyTOF) analysis of peripheral blood mononuclear cells to compare the immunological landscapes in patients with high tuberculous bacillary load PTB infections and LTBI. A total of 32 subjects (PTB [n = 12], LTBI [n = 17], healthy volunteers [n = 3]) were included. Participants with active PTBs were phlebotomized before administering antituberculosis treatment, whereas participants with LTBI progressed to PTB at the time of household screening. In the present study, CyTOF analysis identified significantly higher percentages of mucosal-associated invariant natural killer T (MAIT NKT) cells in subjects with LTBI than in those with active PTB and healthy controls. Moreover, 6 of 17 (35%) subjects with LTBI progressed to active PTB (LTBI progression) and had higher proportions of MAIT NKT cells and early NKT cells than those without progression (LTBI non-progression). Subjects with LTBI progression also showed a tendency toward low B cell levels relative to other subject groups. In conclusion, MAIT NKT cells were substantially more prevalent in subjects with LTBI, particularly those with progression to active PTB.

## 1. Introduction

*Mycobacterium tuberculosis* is a bacterium that commonly causes subacute to chronic tuberculosis (TB) infections that require long-term treatment. Pulmonary TB (PTB) can be transmitted through droplets in the environment and causes either latent TB infection (LTBI) or symptomatic TB, with a triad of subacute fever, weight loss, and night sweats [1]. Because of the significant disease burden and growing concerns about the disease, TB continues to be a global public health problem despite improved international collaboration. LTBI affects a quarter of the population worldwide, posing a significant challenge to the control of TB. In addition to the absence of an effective vaccine, there are difficulties in detecting and treating the slow-growing mycobacterium, and the lengthy treatment involved increases the risk of noncompliance and drug resistance [2]. Of individuals with LTBI harboring *M. tuberculosis*, 5–10% of individuals will develop active TB [3]. Numerous techniques have been used to identify markers for differentiating between LTBI and active TB, as these conditions have distinct clinical implications and require distinct treatment and management strategies [3].

The diverse clinical outcomes of the different patients might partly be a result of the various interactions between innate and adaptive (specific) immunity against *M. tuberculosis* [4]. Recently, novel imaging and single-cell analysis technologies have provided an in-depth pathogenesis of *M. tuberculosis*, including the immune responses [5]. Indeed, high-dimensional mass cytometry, also known as cytometry by time-of-flight (CyTOF), can simultaneously measure many parameters, leading to a huge-spectrum analysis of immune cell populations in several immune-related diseases, including TB [6,7]. Unlike conventional flow cytometry, CyTOF is a variant of flow cytometry in which heavy metal ion markers, rather than fluorochromes, are utilized to identify antibodies [8], leading to the concurrent identification of several protein markers with negligible spillover effects [9].

CyTOF has been used for the immuno–molecular study of TB, including changes in immune profiles during treatment and immune response characterization in the lungs during *M. tuberculosis* control and progression [5,10,11,12]. Because some of the LTBI cases might later progress to the active disease (PTB), CyTOF is also used for a comparative cellular analysis of individuals with LTBI and active PTB [7]. However, there are no reports of single-cell level biomarkers for the progression of LTBI to active PTB. Here, we conducted CyTOF analysis of peripheral blood mononuclear cells (PBMCs) and compared immune cell profiles among subjects with bacteriologically confirmed active PTB, LTBI, and healthy controls.

## 2. Materials and Methods

### 2.1. Study Design and Subjects

**Figure 1:**
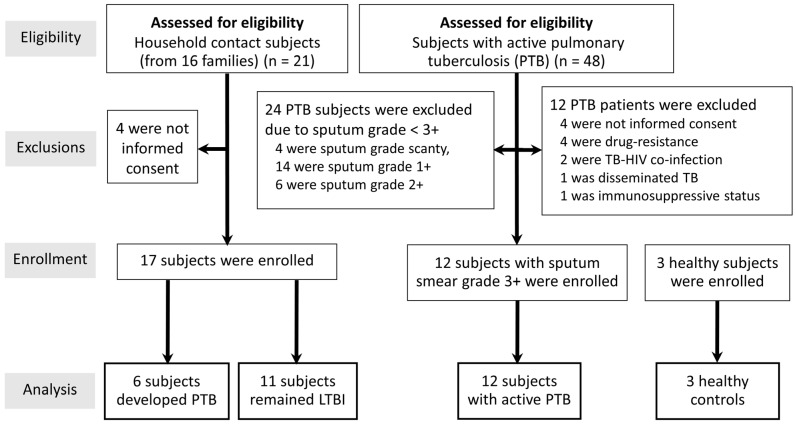
Flowchart of subjects included in the study. LTBI, latent tuberculosis infection; TB-HIV, tuberculosis, and human immunodeficiency virus co-infection.

Adult 48 subjects with naïve active PTB were eligible as the study population. Exclusion criteria consisted of subjects with human immunodeficiency virus (HIV) co-infection, immunosuppression state, pregnancy, and prisoners. Of note, 36 were excluded due to study criteria, as shown in Figure 1. Only 12 active PTB patients with high bacilli loads (sputum smear grade 3+) were enrolled in the study after providing written informed consent at the Hospital for Tropical Diseases, Mahidol University, Thailand, from 1 September 2022 to 30 April 2023. The protocol for this study was approved by the Ethics Committee of the Faculty of Tropical Medicine, Mahidol University (MUTM 2022-053-01).

Eligible subjects with active PTB were asked about subjects with potential LTBI among their household contacts, defined as individuals who had resided in the same house with a subject with active PTB for at least 3 months prior to the diagnosis of PTB in the index subject [13]. 

Subjects with active PTB were diagnosed by either confirmed positive acid-fast bacilli (AFB) staining or positive *M. tuberculosis* culture from sputum, while household contact subjects with LTBI (n = 17) were defined as those with a positive interferon-gamma release assay (IGRA) results, without clinical or radiographic findings compatible with active PTB. All of the household LTBI contacts did not perform sputum culture because they were asymptomatic and unable to produce adequate specimens following induction with nebulized hypertonic saline solution. 

In addition, all participants for the healthy control group (n = 3) were asymptomatic and negative for both IGRA and chest roentgenogram. The healthy control participants were not related to the active TB and household contact LTBI group. The tuberculin skin test was not included as a screening tool for LTBI in this cohort study because it was conducted in an area where TB is endemic. Moreover, the whole Thai population is immunized with Bacillus Calmette–Guérin (BCG) at birth. 

### 2.2. Definitions

#### 2.2.1. Active PTB

Active PTB severity was determined by positive microbiological and abnormal radiographic analyses. 

#### 2.2.2. Quantification of Mycobacterial Load and Severity

Sputum AFB staining was used to quantify mycobacterial load in the sputum. According to the World Health Organization classification, sputum smears were graded as follows: 0, scanty, 1+, 2+, and 3+, using the carbol fuchsin stain (magnification, ×1000) [14]. The severity of PTB was classified as mild or advanced based on whether lesions extended within or beyond one-third of a unilateral lung field on radiographic imaging.

### 2.3. Specimen Collection and Processing

Each subject provided a 10 mL whole blood sample, which was collected into a heparinized tube. All samples were obtained before administering antituberculosis treatment in naïve active PTB (n = 12). Likewise, participants with LTBI progressed to PTB (n = 6), and those without progression to PTB (n = 11) were obtained at the time of household screening. Notably, three healthy controls were collected for blood samples at the enrollment. Collected blood was immediately processed to separate PBMCs and cell-free plasma. Briefly, 4 mL Polymorphprep™ was placed in a 15 mL tube, then 4 mL Ficoll-Plaque carefully layered using a pipette, and 4 mL blood layered on top (blood:Ficoll-Paque: Polymorphprep™ = 4:4:4 ratio). Next, samples were centrifuged at 300× *g* for 30 min and allowed to decelerate without using a brake. Mononuclear cells (top layer) were collected into a new 15 mL tube using a transfer pipette. The collected interface was diluted by adding an equal volume of 1× PBS and centrifuging at 300× *g* for 5 min. The supernatant was aspirated, and cells were resuspended in 1 × PBS before another centrifugation at 300× *g* for 5 min. Cells were then gently mixed with 10% DMSO in fetal bovine serum, transferred to a cryovial tube, and stored under liquid nitrogen until analysis.

### 2.4. Cell Staining and Measurement and CyTOF

Each sample was thawed at room temperature, and 1 M Cell-ID Cisplatin-198Pt (Fluidigm, South San Francisco, CA, USA) was applied over five minutes. Human Fc Receptor Blocking Reagent (Miltenyi Biotec, Bergisch Gladbach, Germany) was added at a dilution of 1:25 for 15 min. To reduce the batch impact, we barcoded each sample with six different anti-CD45 antibodies for 15 min. The samples were fixed for 1 h using 1 mL of Fix/Perm Concentrate (eBioscience, San Diego, CA, USA). 

They were then stained with antibodies (anti-human anti-CD3, anti-CD19, anti-CD14, anti-CD27, anti-CXCR3, anti-CXCR5, anti-CCR6, anti-CD45RA, anti-CD16, anti-CD194-CCR4, anti-TCRgd, anti-CD28, anti-CD127-IL-7Ra, anti-CD56, anti-CD161, anti-CD8, anti-CD4, anti-CCR7, anti-CD25, anti-HLADR, anti-CD20, anti-CD38, anti-CD294, anti-CD57anti-IgD, anti-CD123-IL-3R, anti-CD11c, anti-CD66b, anti-CD45, and anti-CD45RO, Fluidigm, South San Francisco, CA, USA) in permeabilization buffer solution for 30 min with antibodies specific for intracellular markers, followed by 30 min of secondary antibody staining at room temperature with subsequently incubated for 1 h on ice in 1 mL of Maxper Fix and Perm Buffer (Fluidigm, South San Francisco, CA, USA) with 1 M Cell-ID Intercalator-Ir (Fluidigm, South San Francisco, CA, USA). 

The samples were then suspended in a total of 15% EQ Four Element Calibration Beads with Cell Staining Buffer (Fluidigm, South San Francisco, CA, USA). Using a CyTOF Helios system (Fluidigm, South San Francisco, CA, USA), data were collected at a rate of 1000 events per second. Raw FCS data were normalized with CyTOF software version 6.0 (Fluidigm, South San Francisco, CA, USA) using bead-based normalization.

As subjects with higher sputum AFB grade are more likely to transmit the disease and cause active PTB in their contacts than those with a lower sputum AFB grade, as well as exhibiting greater host immune stimulation, only subjects with active PTB and high-grade sputum smear (3+) were tested by CyTOF analysis.

### 2.5. Mass Cytometry

Maxpar Direct Immune Profiling Assay reagent (Fluidigm, South San Francisco, CA, USA) was used to profile 35 immune cell populations from individual peripheral blood mononuclear cells (PBMCs), following the manufacturer’s instructions [15]. Briefly, for mass cytometry, cryopreserved PBMCs were thawed in tepid media containing 10% fetal bovine solution and rinsed. Human TruStain FcX (BioLegend, San Diego, CA, USA) was added to each tube and left for 10 min at ambient temperature to block Fc-receptors. 

After Fc-receptor blocking, PBMCs were transferred directly into a 5 mL vial containing a dried antibody pellet (Fluidigm, South San Francisco, CA, USA) and incubated for 30 min at room temperature. PBMCs were then fixed in 1.6% formalin for 10 min at room temperature. Stained PBMCs were incubated with Cell-ID^TM^ Intercalator-Ir (Fluidigm, South San Francisco, CA, USA) in Maxpar Fix and Perm Buffer (Fluidigm, South San Francisco, CA, USA) at 4 °C for up to 48 h, rinsed twice with Maxpar Cell Staining Buffer (Fluidigm, South San Francisco, CA, USA), and filtered through a 40 µm cell strainer. Between 300 and 500 cell events were acquired per second, with a minimum of 300,000 events acquired in total. The automated software, Maxpar Pathsetter (version 2.0), provided cell counts, percentage calculations, and staining intensity.

Samples from subjects with active PTB (n = 12), LTBI (n = 17), and healthy controls (n = 3) were analyzed by mass spectrometry CyTOF. 

### 2.6. T-SPOT.TB Assay

We used the TB-specific enzyme-linked immunospot assay (T-SPOT.TB) in the study as an early diagnostic method for LTBI. 

The T-SPOT.TB test was performed using a kit (Oxford Immunolyte Ltd., Oxford, UK), according to the manufacturer’s instructions. Briefly, one PBMC was isolated from each sample and incubated with two antigens (ESAT-6 in panel A and CFP-10 in panel B) at 37 °C for 16 to 20 h; the procedure was performed on plates coated with anti-interferon antibodies. Spots were scored using an automated ELISPOT plate reader (AID-Gmb-H, Strasberg, Germany) after the application of an alkaline phosphatase-conjugated secondary antibody and chromogenic substrate. T-SPOT.TB test results were interpreted according to the standard guideline [16].

### 2.7. Statistical Analyses

Data from each group (active PTB (n = 12), LTBI (n = 17), and healthy controls (n = 3) are presented as mean ± standard deviation, and the statistical significance of differences between two (active PTB vs. LTBI) and three groups were assessed by unpaired student *t*-test or one-way analysis of variance (ANOVA) with Tukey’s comparison test, respectively. A *p*-value < 0.05 was considered statistically significant. Statistical analyses and heat map generation were performed using GraphPad Prism 9.5.1 (GraphPad Software, Inc., San Diego, CA, USA).

## 3. Results

### 3.1. Participant Baseline Characteristics

Forty-eight subjects from 36 families were eligible for screening for active PTB (Figure 1). Further, 21 subjects from 16 families met the household contact criteria and were eligible for the LTBI group. Finally, a total of 32 participants (active PTB [n = 12], LTBI [n = 17], and healthy subjects [n = 3]) were enrolled in this study. In the LTBI group, 6 of 17 subjects had progressed to active PTB (latent TB with progression to PTB) by a median (interquartile range [IQR]) 3.2 (1.5–4.4) months. The median (IQR) age of all participants was 46.4 (31.8–67.2) years, and the majority (22 = 69%) were female (Table 1). Of the 12 patients with PTB, all showed positive AFB sputum stain, with no mycobacterium drug resistance, while radiographic examination revealed multilobar involvement in 4 subjects (33%).

### 3.2. CyTOF Analysis Revealed Differential Immune Profiles in Subjects with Active PTB and Those with LTBI

**Figure 2:**
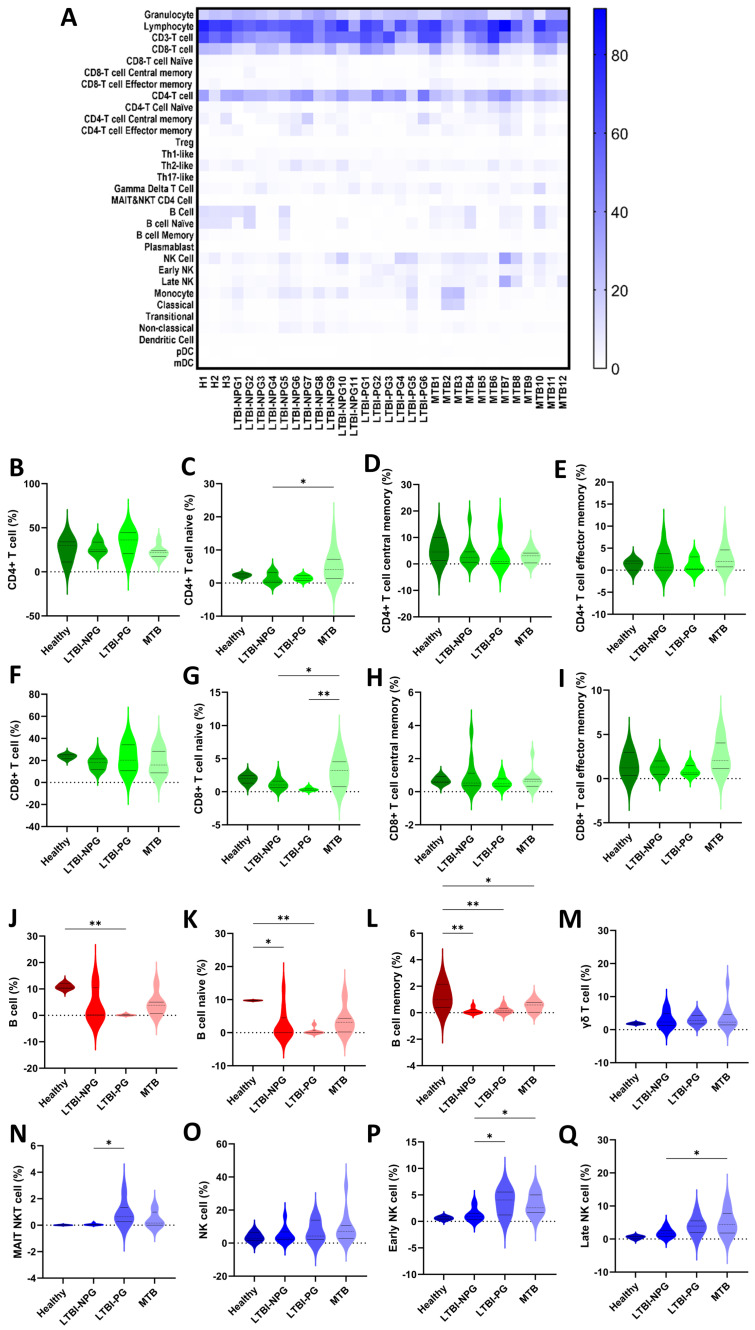
Immune profile characteristics determined by CyTOF analysis. Heatmap showing the percent frequency of immune cell subsets in healthy subjects (H) (n = 3) and subjects with active pulmonary tuberculosis (PTB) (n =12), and latent tuberculosis with non-progression to PTB (LTBI-NP) (n = 11), and latent tuberculosis with progression to PTB (LTBI-PG) (n = 6). (**A**). (**B**–**Q**) Violin plots showing the proportions of T cells (CD4^+^ T cells, CD8^+^ T cells) (green), B cells (red), unconventional T cells (mucosal-associated invariant T (MAIT) cell and γδT cells), and natural killer (NK) cells (blue) subgroups. It is observed that the negative values in the violin plot correspond to estimations of data values as a result of kernel density estimation. Bold and dashed lines in violin plots represent median and quartile values, respectively, for each comparison (* *p* < 0.05 and ** *p* < 0.005). mDC, myeloid dendritic cells; pDC, plasmacytoid dendritic cells.

**Figure 3:**
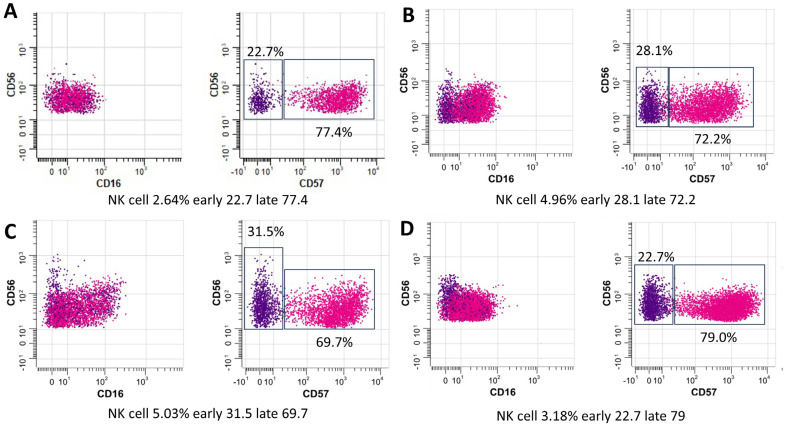
The relative distribution of natural killer (NK) cells (CD14^−^CD3^−^CD123^−^CD66b^−^CD45RA^+^CD56^dim+^CD57^−>+^) among experimental groups: Healthy. (**A**), active PTB (**B**), LTBI progression (**C**), LTBI non-progression (**D**). The plots represent individuals of each group pooled (blue and red represent early NK cells (CD56) and late NK cells (CD57), respectively).

We assessed the immune profiles of all enrolled subjects (n = 32) by CyTOF analysis of PBMC samples. Following our protocol, it has been optimized for an average of 3 × 10^6^ cells per sample. There was a clearly increased percentage of staining for mucosal-associated invariant T cells-natural killer T (MAIT NKT) cells in subjects with LTBI relative to those with active PTB and healthy controls. A heatmap showing variations in immune cell subsets among individuals with PTB, LTBI, and healthy subjects is presented in Figure 2A. Analysis of the diversity of T cells, B cells, and their subpopulation (Figure 2B–Q) indicated high percentages of lymphocytes, granulocytes, CD3^+^ T cells, CD4^+^ T cells, and CD8^+^ T cells in all groups. Higher proportions of B cells, naïve B cells, and memory B cells were detected in healthy subjects. Further, the proportions of MAIT NKT cells and early NK T cells differed significantly between subjects in the LTBI non-progression and LTBI progression groups. Notably, subjects with LTBI progression showed higher proportions of both MAIT NKT and early NK T cells (Figure 2N,P), with a tendency toward a higher percentage of late NK T cells relative to those in the LTBI non-progression group (Figure 2P). In addition, the proportion of CD16^+^CD56^dim^CD57^+^ early NK cells among PBMCs was significantly higher in subjects with LTBI progression to PTB than in those with LTBI non-progression to PTB (median: 31.5% vs. 22.7%, *p* = 0.01) and active PTB (median: 31.5% vs. 28.1%, *p* = 0.03) (Figure 3).

**Figure 4:**
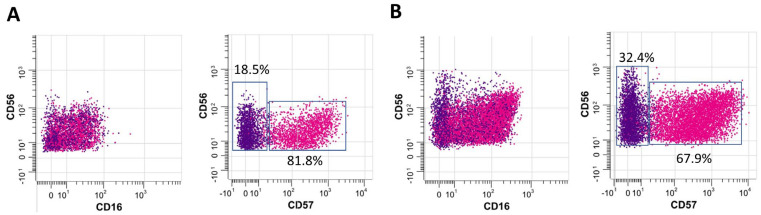
Comparison of the relative distribution of natural killer (NK) cells (CD14^−^CD3^−^CD123^−^CD66b^−^CD45RA^+^CD56^dim+^CD57^−>+^) between participants who were (**A**) non-responders (smear positive, n = 4) and (**B**) treatment responders (smear-negative, n = 14). The plots represent individuals of each group pooled (blue and red represent early NK cells (CD56) and late NK cells (CD57), respectively).

Next, we applied CyTOF to comprehensively characterize the relationship between circulating immune cell populations in individuals with active PTB (n = 18) (12 from active PTB and 6 from LTBI progression) and outcomes after 2-months of treatment with standard antituberculosis regimens [2 months of HRZE (isoniazid, rifampicin, pyrazinamide, and ethambutol), followed by 4 months of HR]. Interestingly, we found that the initial proportion of CD16^+^CD56^dim^CD57^+^ early NK cells among PBMCs was higher in participants who were treatment responders (smear-negative, n = 14) than in non-responders (smear positive, n = 4) (median: 32.4% vs. 18.5%, *p* = 0.01) (Figure 4).

According to an analysis of B cell populations, there was a consistently significant decrease in the total B cell population and B cell subpopulations (naïve and memory B cells) in subjects with active PTB relative to healthy controls. Indeed, the percentage decrease was approximately 7% and 4% in active PTB and LTBI, respectively. Interestingly, subjects with LTBI progression showed a tendency toward lower B cell levels compared with other groups (Figure 2J–L). Proportions of other immune cells, including dendritic, T-helper (Th) 1-like, Th2-like, and Th17-like cells, were comparable among groups.

## 4. Discussion

We explored the immune landscape characteristics of subjects with latent TB (LTBI) and active TB (PTB; sputum smear grade 3+) using CyTOF (mass cytometry) analysis, which provides several advantages over the regular flow cytometry, including the ability to simultaneously analyze multiple markers and provide more comprehensive information [17]. 

### 4.1. NK Cells and NK T Cells, Important Innate Immune Cells against Tuberculosis

Here, CyTOF demonstrated higher proportions of CD4^+^, CD8^+^, and late NK T cells in patients (PTB and LTBI) than the healthy controls. Indeed, CD8^+^ cells are the major adaptive immunity responsible for the control of intracellular organisms, including *Mycobacterium tuberculosis*, that are enhanced by helper T cells (CD4^+^ cells). In parallel, NK T cells are innate immune cells activated by microbial glycolipid [18], a major part of the mycobacterium cell envelope [19], which are developed into late NK T cells with the maturation of several microbicidal molecules [20]. Hence, the higher proportions of CD4^+^, CD8^+^, and late NK T cells in patients with active TB might indicate an active immune response against the high abundance of organisms. Meanwhile, subjects in the LTBI non-progression group had lower MAIT cells, NKT cells, and NK cells than those in both the LTBI progression and PTB groups. Accordingly, these innate immune cells (MAIT cells, NKT cells, and NK cells) are important for mycobacterial control, and the lower number of these cells in LTBI non-progression compared with the conditions with the higher microbial abundance (LTBI progression and PTB groups) might be one indirect indicator for the lower microbial abundance. On the other hand, the proportion of CD16^+^CD56^dim^CD57^+^ early NK cells among PBMCs was significantly higher in subjects with LTBI progression to PTB than in those in the LTBI non-progression group. As such, CD16 (Fc gamma receptor III) indicates, at least in part, the microbicidal property through antigen-dependent cell cytotoxicity (ADCC) and reduced CD56 (the adhesion molecule) with positive CD57 implies the senescent of these cells [21]. Thus, the higher CD16^+^CD56^dim^CD57^+^ early NK cells in LTBI progression compared with the active TB (PTB) might indicate the activity of cells with ADCC property that enable organismal control in LTBI progression, and a reduction in these cells might correlate with the TB disease activities similar to a previous study [22]. In addition, the subjects with LTBI progression had significantly higher proportions of MAIT NKT cells than those who did not progress to PTB. The importance of increased MAIT NKT cells in LTBI progression could be explained by the involvement of these cells in antimycobacterial immunity. Unlike other immune cells activated in response to mycobacteria (i.e., HLA-E-restricted T cells and TCRγδ T cells), MAIT NKT cells are highly equipped with the cytotoxic molecules via granzyme B activity and contribute to the early response against mycobacteria [23]. Moreover, the gene expression profiles of activated NK cells and conventional CD8^+^ T cells overlap with those of MAIT cells, further supporting a role for MAIT NKT cells as a functional link between innate and adaptive immunity [24]. However, at the later time point, failure of mycobacterial control by MAIT NKT cells might lead to the recruitment of early (CD16^+^CD56^dim^) and late (CD16^−^CD56^bright^) NK cells (Figure 2N,P,Q) as the supportive groups of the cells for controlling the mycobacteria. It seems that the NKT cells, a heterogeneous group of T cells that share properties of both T cells and NK cells, might have an initial impact on the mycobacterial control first, followed by the NK cells. This hypothesis is supported by the significantly lower proportions of advanced NK cell differentiation in subjects with PTB treated with anti-TB regimens [25]. Interestingly, while NK cell frequencies were similar among all groups (Figure 3), significantly increased CD57 expression was observed in NK cells from subjects with LTBI. Accordingly, CD16^+^CD56^dim^CD57^+^ early NK cells are potential diagnostic markers for LTBI, as well as a possible prognostic marker following treatment; further studies are warranted.

### 4.2. Impacts of B Cells and Th Cells in Pulmonary Tuberculosis

Although B cells and antibodies contribute to immunity against mycobacteria, the impacts of B cells against tuberculosis are still inconsistent [26,27,28,29]. Here, the total B cell population and B cell subpopulations (naïve and memory) were consistently and significantly lower in subjects with active PTB than those in healthy subjects (Figure 2J–L), with the converse distribution detected for T and NK cells (Figure 2A). The higher B cell populations in healthy volunteers than the patients (active and inactive TB) elaborated on the potential role of B cells in protective immunity against mycobacterium infection, possibly through antibody production and the effective antigen-presenting cells for CD4^+^ T cell stimulation [26]. Indeed, passive transfer with the antibodies against mycobacterial antigens demonstrates protection against tuberculosis [30], and several B cell-producing cytokines also have some impacts on viral control [31]. For T cell populations, T-helper 1 (Th1) cells control mycobacteria by secreting IFN-γ and triggering antimycobacterial responses of macrophages [32,33,34], while Th17 cells are responsible for neutrophilic inflammation, lung tissue damage [35], and improved survival [36]. While Th 17 cells demonstrate multifaceted functions during TB infection (microbial control versus lung injury), Th1 activation seems to be mainly beneficial through microbial control [37], and Th 2 induction might enhance the complications (fibrosis and cavitation) partly through IL-4 production [38]. However, the comparable abundances of Th1, Th2, and Th17 cells between TB cases (LTBI and PTB) and healthy control implied less influence of Th cells on mycobacterial control compared with B cells and NK T cells. 

### 4.3. Clinical Translation, Limitations, and Future Directions

For the clinical translation, some of the markers might be interesting candidates for the monitoring of disease progression in patients with latent TB, leading to more frequent follow-ups and rapid treatment. For example, patients with latent TB with elevated CD16^+^CD56^dim^CD57^+^ early NK cells or MAIT NK cells might need closer monitoring than latent TB without this biomarker because of the higher possibility of developing active TB. Additionally, the monitoring of B cell abundance might be helpful for monitoring TB disease activity as the elevation of B cell subsets during treatment of active TB might have a better prognosis than the patients with reduced B cells. More studies for using these parameters for the novel TB biomarkers are interesting. Several limitations need to be mentioned. First, the relatively small number of participants might limit the statistical differences between active TB and latent TB (LTBI) despite the benefits of CyTOF analysis in studies with small numbers of subjects [39]. Although our protocol has been optimized for adequate cells per sample for the analyses [40], as well as a consistent number across samples, an increased number of participants will demonstrate a more solid conclusion. Notably, the analytic methods in this study were based on the separate t-SNE algorithm but not the pool samples in the same study group. Then, the analysis using the pool samples might produce different results and conclusions. Further studies are needed. Second, no batch effect correction (removal of the uncorrelated variabilities of the procedures) has been applied across the samples in the present study. However, there is no variation in the type of machine and the technicians in our study. Third, the impact of different BCG vaccinations was not considered in this study. Although BCG immunization causes immune responses against tuberculosis (variation in interleukin-17 and IFN-γ) [41], nearly all Thai citizens received BCG vaccination following the Thailand Expanded Programme on Immunization (EPI). The evaluation of BCG protection levels might improve the understanding of immune responses against mycobacteria. Fourth, the cell surface markers derived from cryopreserved PBMCs that were used in the current study might be different from the fresh samples [42]. Then, studies with larger sample sizes, batch effect correction, BCG determination, and further exploration of specific immune cell populations are warrants for a more solid conclusion. 

## 5. Conclusions

In summary, our CyTOF analysis of PBMCs revealed comparable immune cell populations in subjects with active TB and LTBI, with the exception of MAIT NKT cells, which were substantially more prevalent in subjects with LTBI who progressed to active TB. In addition to MAIT NK T cells, B cells, rather than Th cells, have the potential to be effective markers for distinguishing between latent TB infection and active TB. Further studies are warranted. Our analysis of the circulating immune landscape improved knowledge of the immunopathogenesis of TB and provides prospective targets for treatment, as well as a potential TB control strategy for improving LTBI diagnosis by enhancing case detection and thereby containing disease spread by interrupting transmission.

## Figures and Tables

**Table 1:** Characteristics of subjects with active pulmonary tuberculosis (PTB), latent tuberculosis infection (LTBI), and healthy Controls.

Parameter (s)	PTB (n = 12)	LTBI (n = 17)		Healthy (n = 3)
		Non-Progression to PTB (n = 11)	Progression to PTB (n = 6)	
Age, years	48.9 ± 15.1	30.6 ± 7.7	43.4 ± 9.8	33.7 ± 9.4
Female, n (%)	8 (66.7)	7 (63.6)	6 (100)	1 (33.3)
Duration from onset of symptoms (days)	18 ± 9	N/A	N/A	N/A
Fever, n (%)	12 (100)	N/A	N/A	N/A
Weight loss, n (%)	12 (100)	N/A	N/A	N/A
Cough, n (%)	12 (100)	N/A	N/A	N/A
Sweating, n (%)	11 (91.7)	N/A	N/A	N/A
Lymphadenopathy, n (%)	8 (66.7)	N/A	N/A	N/A
Chest radiographic distribution				
Lesions localized within one-third of the unilateral lung zone	5 (41.7)	N/A	N/A	N/A
Lesions localized over one-third of the unilateral lung zone	3 (25.0)	N/A	N/A	N/A
Lesion enhanced as bilateral lung zones	4 (33.3)	N/A	N/A	N/A

Data are presented as n (%) or median ± standard deviation unless otherwise stated. N/A, not applicable.

## Data Availability

The data that support the findings of this study are available from the corresponding author upon reasonable request.
